# Cross-sectional association between medical expenses and intellectual activity in community-dwelling older adults

**DOI:** 10.1186/s12199-017-0672-1

**Published:** 2017-08-25

**Authors:** Kimiko Tomioka, Norio Kurumatani, Hiroshi Hosoi

**Affiliations:** Nara Prefectural Health Research Center, Nara Medical University, Shijo-cho 840, Kashihara city, Nara 634-8521 Japan

**Keywords:** Community-dwelling elderly, Health behaviors, Higher-level functional capacity, Intellectual activity, Medical expenses

## Abstract

**Background:**

Little is known concerning the lifestyle habits and health conditions in community-dwelling elderly who do not get medical care. We investigated the cross-sectional association between medical expenses (ME) and intellectual activity (IA) in community-dwelling older Japanese.

**Methods:**

Self-administered questionnaires were mailed to all residents born between 1945 and 1949 and covered by A City’s medical insurance system (*n* = 19,354). Independent variables including health behaviors, oral health, social capital, neighborhood environment, and physical and mental functioning were included in the questionnaires. Medical fee receipts were used to evaluate ME for fiscal 2014, and respondents were classified into no, low, medium, and high ME groups. Higher-level functional capacity was evaluated using the Tokyo Metropolitan Institute of Gerontology Index of Competence, which is comprised of three subscales: instrumental activities of daily living, IA, and social role. Poisson regression models were used to examine the association of ME with IA, with the low ME group as reference.

**Results:**

Questionnaires were returned by 12,747 individuals (response rate 65.9%). The no ME group had the lowest response rate, the worst lifestyle behaviors, and the lowest social capital, but no problems with neighborhood environment. Higher-level functional capacity, especially IA, was reduced in both the high ME and no ME groups. After adjustments for age, gender, health insurance, accessibility to public facilities in their residential area, family size, body mass index, and physical and mental functioning, the prevalence ratio (PR) for impaired IA lost its significance in the high ME group (PR 0.97, 95% confidence interval 0.90–1.05), but remained significant in the no ME group (1.19, 1.08–1.31). After additional adjustments for health behaviors (i.e., health checks, smoking, fitness, and dietary variety), the PR of the no ME group was attenuated towards the null (1.08, 0.98–1.20).

**Conclusions:**

Community-dwelling elderly who did not seek medical treatment were indifferent to health surveys and health-promoting behaviors, and undesirable health behaviors were a possible determinant of their impaired IA. Further longitudinal research is needed to confirm the causal associations.

**Electronic supplementary material:**

The online version of this article (10.1186/s12199-017-0672-1) contains supplementary material, which is available to authorized users.

## Background

With the advancement of medicine and improvement of public health, average life expectancy has been increasing in both advanced countries and developing countries, meaning they must cope with societal aging. As of October 2015, the population aging rate in Japan is the world’s highest, at 26.7% [1], and is proceeding at a much greater speed in Japan than in European and other western countries [1]. Ordinarily, elderly people tend to have many more ailments than younger people, so medical expenses (ME) naturally become much higher in aging populations [2]. In a super-aging society like Japan, the issue of suppressing health-care expenditures is urgent. Some municipalities in Japan have tested schemes such as giving a reward to those who do not receive health insurance treatment in an effort to suppress ME [3]. Therefore, it is necessary to investigate the lifestyle habits, health conditions, and higher-level functional capacities of the community-dwelling elderly who do not undergo medical treatment.

Higher-level functional capacity refers to a capacity that is beyond the basic activities of daily living (BADL) such as walking, having meals, taking a bath, and excreting. In other words, higher-level functional capacities are those required for elderly people to live independent lives in their community and include instrumental activities of daily living (IADL), intellectual activity (IA), and social role [4]. Maintaining these higher-level functional capacities signifies healthy aging. Past research on Japanese community-dwelling elderly has noted significant and independent associations between higher-level functional capacity and health behaviors such as smoking, fitness habits, and hours of sleep [5]; dietary habits such as dietary diversity [6] and animal protein intake [7]; oral health behaviors such as the use of extra cleaning devices [8]; oral function such as chewing ability [9]; social capital such as social participation [10]; and physical and mental functioning such as self-rated health [11], depression status [12], and cognitive performance [13]. On the other hand, significant factors involving increased ME include cardiovascular risk factors [14], obesity [15, 16], and less walking [17] among Japanese local residents; dietary diversity among Taiwanese elderly [18]; and meat consumption among Europeans and North Americans [19]. However, to the best of our knowledge, the relationships of higher-level functional capacity and medical care utilization on local-dwelling elderly are not known. To extend healthy life expectancy and realize a sustainable social security system in a super-aging society, it is meaningful to investigate the association between the extent of medical care utilization and higher-level functional capacity.

The aim of this study was to examine the cross-sectional relationship between ME and higher-level functional capacity in Japanese aged 65–70 years, using community-based data.

## Methods

### Study population

Data were obtained from the Nara Data Health Survey, a cross-sectional population-based survey conducted by A City in Nara prefecture. Nara prefecture is positioned roughly in the center of Japan and is a landlocked municipality surrounded by the prefectures of Osaka, Kyoto, Wakayama, and Mie. During and after a high-growth period, Nara prefecture has become a bedroom community for the cities of Osaka and Kyoto. According to census figures [20], the prefecture with the highest rate of employed persons who are working in other prefectures is Nara. In October 2015, questionnaires were distributed to all people who satisfied the following three conditions: (1) they lived in A City on March 31, 2015, (2) they were born between 1945 and 1949, and (3) they were covered by the National Insurance, medical insurance for later-stage elderly (younger elderly people can be covered if their basic activities of daily living are low due to suffering from certain disorders), or livelihood subsidies, and the coverage period by one of the above was 1 year or longer as of March 31, 2015. As of January 1 in 2015, these three insurance systems covered an estimated 65% of all citizens born from 1945 to 1949.

### Medical expenses

The total ME of each subject was calculated based on medical fee receipts for the 12 months from April 2014 through March 2015. The total ME for fiscal 2014 was defined as each subject’s ME and expressed in Japanese yen; according to foreign exchange rates of April 1, 2016, 0.90 US dollars or 0.78 euro equaled 100 Japanese yen. Based on their ME, participants who submitted questionnaires were categorized into two groups: those who did not have ME (hereafter, “no ME group”) and those who had ME. The ME of men was significantly higher than those of women (Mann-Whitney test, *p* < 0.001), and the ME of older people (ages 68 to 70 years at the time of the survey) was significantly higher than that of younger people (ages 65 to 67 years at the time of the survey) (Mann-Whitney test, *p* < 0.001). Study participants with ME were then categorized into four groups based on gender and age (males 65 to 67 years old, males 68 to 70 years old, females 65 to 67 years old, and females 68 to 70 years old), and each group was separated into tertiles based on ME. Study participants were then categorized into four groups: no ME, low ME, medium ME, and high ME.

### Health behaviors

Assessments of health checkups were based on medical fee receipts, and the participants were dichotomized into having health checkups or having no health checkups during the 2014 fiscal year. Lifestyle habits were assessed by a self-administered questionnaire. Smoking habit was defined as never-smoker, ex-smoker, or current smoker. Number of years of smoking was classified as zero, 1–14, 15–29, or ≥ 30. Frequency of alcohol consumption was classified as none, social, occasional, or daily. Frequency of exercise (hours per week) was classified as < 1, 1–2, 3–4, or ≥ 5. Walking time (minutes per day) was classified as < 30, 30–59, or ≥ 60 [21]. Questions about dietary habits were measured using the dietary variety score (range 0–10) [22]; a higher score indicates a higher level of dietary variety. The participants were classified into tertiles according to their dietary variety score: T1 (scores of 0–2) indicated low variety, T2 (scores of 3–4) indicated medium variety, and T3 (scores of 5–10) indicated high variety.

### Oral health

Oral health was assessed from a self-administered questionnaire that was based on previous studies [8, 9, 23–25] and consisted of two parts: dental health behaviors and oral function. For dental health behaviors, we assessed the use of extra cleaning devices such as interdental brushes or dental floss (yes or no), the frequency of daily tooth brushing (three or more times a day, twice a day, or less than twice a day), and the frequency of bedtime tooth brushing (daily, sometimes, or almost never). Assessment of oral function included having difficulty in chewing hard foods (yes or no), choking on tea or soup (yes or no), having dry mouth (yes or no), and the use of dentures (yes or no).

### Social capital

To measure social capital, we consulted previous studies [26–28] and decided to evaluate social participation, social support, and social networks through a questionnaire. Social participation was categorized into six types: volunteer groups, sports groups, hobby groups, senior citizens’ clubs, neighborhood community associations, and cultural groups. The number of groups in which respondents participated was classified into none, one, two, and three or more. Social support was measured by the question, “How many people do you have that you can talk to if you feel troubled?: nobody (none), one person, two to three persons, four or more persons.” Social networking was measured by the question, “How often do you meet your friends and acquaintances?: nearly every day, several times per week, several times per month, several times per year, almost never (none).”

### Neighborhood environment

Because a prior study suggested that environmental factors can either facilitate or hamper medical care utilization [29], we assessed neighborhood environment using the Japanese version of the International Physical Activity Questionnaire Environment Module (IPAQ-E) [30]. To evaluate neighborhood environment related to ME, we used two items from IPAQ-E: access to public facilities including medical institutions and access to public transport.

### Physical and mental functioning

Assessment of physical and mental functioning was done by the self-administered questionnaire. Individuals with impaired BADL were defined as those who reported needing help to perform any of five BADL items: eating, dressing, bathing, going to the toilet, and walking indoors. Self-rated health was assessed through the single questionnaire item, “How is your health in general? Is it very good, rather good, rather poor, or very poor?” and was dichotomized into good (very good/rather good) and poor (rather poor/very poor). Health-related quality of life (QOL) was assessed using the Japanese version of the 8-item Short-Form Health Survey (SF-8) [31]. The SF-8 measures eight domains of health status and can be used to calculate two summary scores: the Physical Health Component Score (PCS) and the Mental Health Component Score (MCS), with higher scores indicating better QOL. The participants were classified into tertiles according to their PCS/MCS: T1 indicated low PCS/MCS, T2 indicated medium PCS/MCS, and T3 indicated high PCS/MCS. Sleep disturbance was evaluated using the Pittsburgh Sleep Quality Index (score range 0–21) [32], and those with a score of > 5.5 were considered to have sleep disturbance issues. Depression was evaluated using the 5-item short form of the Geriatric Depression Scale (score range 0–5) [33], and those with a score of ≥ 2 were considered to have depression. Cognitive functioning was evaluated using the Cognitive Performance Scale (score range 0–6), and those with a score of ≥ 1 were considered to have poor cognitive functioning [34].

### Higher-level functional capacity

Higher-level functional capacity was assessed using the Tokyo Metropolitan Institute of Gerontology Index of Competence (TMIG-IC) [35]. The TMIG-IC was developed to measure higher-level competence on the basis of Lawton’s hierarchical model of behavioral competence [4]; the first five questions are about IADL (scores 0–5), the subsequent four questions are about IA (scores 0–4), and the final four questions are about social role (scores 0–4). A higher score indicates a higher functional level of higher-level competence. The reliability and validity of the TMIG-IC have been established [36]. For total score (score range 0–13), participants with a score of ≤ 11 were defined as having impaired higher-level functional capacity. This cutoff value was based on a previous report that the variation of 1 point for total score on the TMIG-IC was regarded as a possible measurement error [37]. For subscale scores, participants with a score of 1 or more below the respective full mark were defined as having impaired capacity, i.e., a score of ≤ 4 for IADL and a score of ≤ 3 for IA and social role [38]. Because social role has a conceptual configuration similar to social capital, which was one of the independent variables in this study [4], we thought that selecting social role as a dependent variable was not appropriate due to the problem of multicollinearity. Additionally, because prior studies have revealed that impaired IA can predict a decline in IADL in community-dwelling older people [10, 11, 39], we considered that IA was a more effective indicator than IADL of higher-level functional capacity in the elderly. Therefore, we decided to adopt IA as our main outcome. The IA subscale is defined as “activity emanating from the motivation to explore” [4] and is composed of four questions [35]: “Are you able to fill out forms for your pension?,” “Do you read newspapers?,” “Do you read books or magazines?,” and “Are you interested in news stories or programs dealing with health?”

### Covariates

Age, gender, type of health insurance, accessibility to public facilities in residential area, family size, and body mass index (BMI) were collected as covariates. Information on age, gender, health insurance, and residential area was provided by the city hall, and family size, BMI, and individual-level accessibility to public facilities were collected from a questionnaire. Health insurance was categorized as National Insurance, medical insurance for later-stage elderly, or livelihood subsidies. Family size was categorized as one (i.e., living alone), two, three, or four or more persons. The BMI (kg/m^2^) was calculated as weight (kg) divided by height squared (m^2^) and categorized as normal (18.5–< 25.0), underweight (< 18.5), or obese (≥ 25.0) based on the definition by the Japan Society for the Study of Obesity [40]. For accessibility to public facilities, area-level facilities instead of individual-level facilities were set as covariates with the purpose of adjusting for local characteristics. In detail, A City was divided into 50 areas based on sphere of life, and the rate of participants who answered “poor” on accessibility to public facilities in each area was calculated based on accessibility to public facilities as evaluated with IPAQ-E [30]. The calculated rate of those who answered “poor” was then divided in two at the median value and classified into the areas of either “good” or “poor” based on accessibility to public facilities. As a result, 7421 residents in 25 areas were categorized as living in “good” areas, and 5326 residents in 25 areas were categorized as living in “poor” areas.

### Statistical analysis

A trend test to detect an increased prevalence of participants’ characteristics among the four ME groups was performed using the Cochran–Armitage test. To examine the relationship between ME as an independent variable and IA as a dependent variable, a prevalence ratio (PR) and a 95% confidence interval (CI) for impaired IA (i.e., IA score ≤ 3) were calculated using Poisson regression analyses with robust variance estimators [41]. Participants in the low ME group were set as the reference category. Age (continuous), gender, health insurance, accessibility to public facilities in their residential area, family size, and BMI were adjusted for in Model 1. In addition, self-rated health, health-related QOL (PCS and MCS), sleep disturbance, depression, and cognitive functioning (i.e., physical and mental functioning) were adjusted for in Model 2; BADL was not included in Model 2 because all people with medical insurance for later-stage elderly were limited in BADL. To investigate which factors explained impaired IA in the no ME group, health behaviors, oral health, or social capital were added to each model from Model 3 to Model 5, and changes in the PR estimates for impaired IA were explored. When the PR for impaired IA was attenuated towards the null and underwent a major change, it was considered that the additional factor was a more potential determinant of impaired IA in the no ME group than were other factors. Model 3 added participation in health checks, current smoking status, frequency of exercise, and dietary variety (i.e., health behaviors) to the variables in Model 2. Model 4 added the use of extra cleaning devices, the frequency of bedtime brushing, difficulty with hard foods, and the use of dentures (i.e., oral health) to the variables in Model 2; frequency of daily brushing was excluded from Model 4 because of a significant association with the frequency of bedtime brushing (Spearman’s coefficient = 0.56). Model 5 added social participation, social support, and social networks (i.e., social capital) to the variables in Model 2.

Statistical analyses were performed using SPSS version 24.0 J (IBM SPSS Inc., Chicago, USA) with a significance level of 5% (two-sided test).

## Results

**Fig. 1:**
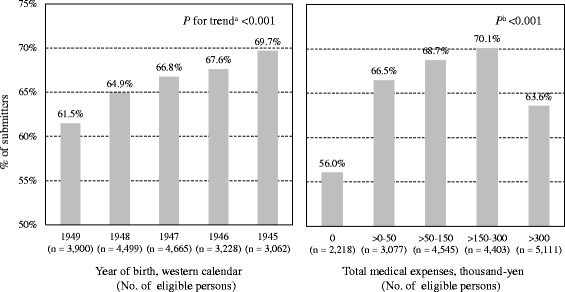
Percentage of individuals who submitted the questionnaire by year of birth and total medical expenses for the 2015 fiscal year. Superscript “a” indicates Cochran-Armitage test, and superscript “b” indicates chi-square test

Among the 19,354 eligible individuals, 12,747 returned the questionnaires (response rate 65.9%). Basic attributes of individuals with or without submission of the questionnaire are shown in Table 1 and Fig. 1. Compared to individuals who submitted the questionnaire, non-submitters were more likely to be male, young, and dependent in BADL. Regarding ME, the response rate was lowest for residents who had no ME; the second lowest response rate was for those who had the highest ME.

**Table 1:** Basic attributes of individuals with or without submission of the questionnaire

Basic attributes	Submitters of the questionnaire (*n* = 12,747)		Non-submitters of the questionnaire (*n* = 6607)		*P* ^*a*^
	*n*	%	*n*	%	
Gender					
Male	5413	42.5	3073	46.5	< 0.001
Female	7334	57.5	3534	53.5	
Health insurance					
National Insurance	12,321	96.7	6295	95.3	0.082
Medical insurance for later-stage elderly	109	0.9	73	1.1	
Livelihood subsidies	317	2.5	239	3.6	
Basic activities of daily living dependency^b^					
Absent	12,480	97.9	6340	96.0	< 0.001
Present	267	2.1	267	4.0	

^a^Differences between submitters and non-submitters were analyzed using the chi-square test

^b^Individuals who were certified as in need of support/care-level as per the national long-term care insurance system

Regarding the characteristics of the study population (see Additional file 1), males were more likely to be older, live with more persons, be obese, have health checkups, be smokers and daily drinkers, exercise, have difficulty in chewing hard foods, and suffer depression and poor cognitive functioning. Females were more likely to have National Insurance, be underweight, have high dietary variety, have good dental health behaviors, have good self-rated health and high MCS, suffer sleep disturbance, have a high level of social capital, and preserve higher-level functional capacity.

Table 2 shows the characteristics of the study population by four groups based on ME. Participants with more ME were more likely to be obese, have less time to walk, have more difficulty swallowing, have dry mouth, report poor physical and mental functioning, and perceive the environment of their neighborhood as poor. In contrast, the no ME group had the worst scores in health behaviors other than walking time, dental health behaviors, chewing ability, and social capital across the four groups. Additionally, the no ME group had the same level of impaired higher-level functional capacity as the high ME group.

**Table 2:** Characteristics of the study population by medical expense category

	*n* ^a^	Medical expense category				P for trend^b^	*P* value^c^
		None	Low	Medium	High		
Medical expenses, median, Japanese yen per year							
Men aged 65–67 years	2439	0	48,360	184,390	536,820		
Men aged 68–70 years	2974	0	60,660	198,815	569,180		
Women aged 65–67 years	3611	0	37,710	151,105	384,020		
Women aged 68–70 years	3723	0	45,080	163,675	425,820		
Basic attributes							
Health insurance: other than NI	12,747	0.8%	1.1%	2.2%	7.5%	​***	***
Family size: one (i.e., living alone)	12,600	17.5%	14.8%	13.5%	15.3%		
Obese: BMI ≥ 25.0	12,598	14.8%	13.5%	18.9%	24.7%	***	***
Health behaviors							
Health checks: non-participation	12,747	84.4%	58.9%	55.0%	68.1%		***
Current smokers	12,462	23.3%	14.2%	11.4%	10.6%		***
Daily drinkers	12,639	25.9%	24.6%	23.7%	18.7%		***
Frequency of exercise: < 1 h/week	12,527	46.8%	35.8%	36.2%	44.7%		
Walking time: < 1 h/day	12,557	49.7%	51.9%	53.5%	61.3%	***	***
Dietary variety: low	12,585	40.7%	35.4%	35.4%	39.8%		
Oral health							
Use of extra cleaning devices: no	12,283	55.9%	42.6%	40.6%	44.4%		***
Daily brushing frequency: < 2 times/day	12,280	29.4%	20.7%	20.4%	23.8%		***
Bedtime brushing frequency: not daily	12,279	29.9%	19.2%	19.5%	24.5%		***
Having difficulty in chewing hard foods	12,279	27.0%	19.6%	18.5%	24.3%		
Choking on your tea and soup	12,266	13.8%	18.1%	18.1%	23.5%	***	***
Having dry mouth	12,254	16.8%	20.1%	22.7%	29.3%	***	***
Users of dentures	12,230	39.2%	36.5%	37.1%	41.0%		
Social capital							
Social participation: none	12,534	37.9%	26.2%	27.3%	35.0%		
Social support: nobody	12,430	10.1%	6.7%	7.1%	7.7%		**
Social networks: none	12,439	11.1%	7.9%	7.7%	11.4%		
Neighborhood environment							
Access to public facilities: poor	12,484	33.8%	34.5%	35.3%	37.7%	**	*
Access to public transports: poor	12,463	8.3%	7.3%	8.7%	9.5%	**	
Physical and mental functioning							
Basic activities of daily living: impaired	12,586	0.5%	0.3%	0.8%	4.4%	***	***
Self-rated health: rather poor or very poor	12,484	5.0%	6.3%	12.5%	33.5%	***	***
Health-related QOL: low PCS	12,505	19.1%	23.6%	32.2%	49.1%	***	***
Health-related QOL: low MCS	12,505	28.7%	28.8%	31.7%	41.1%	***	***
Sleep disturbance: present	12,283	19.6%	22.8%	29.5%	40.0%	***	***
Depression: present	12,437	21.4%	17.8%	21.4%	29.7%	***	***
Cognitive functioning: poor	12,580	11.8%	10.5%	12.2%	19.0%	***	***
Higher-level functional capacity							
TMIG-IC: a score of 11 or less	12,425	38.3%	30.8%	31.3%	39.2%		

*MCS* Mental Component Summary, *ME* medical expenses, *NI* National Insurance, *PCS* Physical Component Summary, *QOL* quality of life, *TMIG-IC* Tokyo Metropolitan Institute of Gerontology Index of Competence

^*^ *p* < 0.05; ^**^ *p* < 0.01; ^***^ *p* < 0.001

^a^Number of valid responses

^b^Dose-response relationship between more ME and more persons corresponding to the item was analyzed using the Cochran-Armitage test

^c^Difference between the no ME group and the high ME group was analyzed using Fisher’s exact test

The prevalence of impaired higher-level functional capacity by subscale was evaluated (see Additional file 2). Participants with impaired IADL were observed most frequently in the high ME group, while for IA and social role, those with impaired capacity were equally represented in the high ME group and the no ME group.

Table 3 shows the PRs for impaired IA associated with ME. In the crude model, the high ME group and the no ME group were significantly associated with impaired IA: PR = 0.98 (95% CI = 0.91–1.06) for the medium ME group, 1.26 (1.17–1.35) for the high ME group, and 1.27 (1.16–1.40) for the no ME group, compared to the low ME group. These significant associations were unchanged after adjustment for covariates (Model 1). However, after additional adjustment for physical and mental functioning (Model 2), the association of the high ME group with impaired IA disappeared, and a significant association remained only for the no ME group: PR = 0.97 (95% CI = 0.90–1.05) for the high ME group and 1.19 (1.08–1.31) for the no ME group. To explore explanatory factors for impaired IA among the no ME group, we added health behaviors, oral health, or social capital to each model from Model 3 to Model 5. In Model 3, where the data were adjusted for health behaviors in addition to the variables in Model 2, the PR of the no ME group dropped to 1.08 (95% CI = 0.98–1.20), and significance disappeared. After adjusting for oral health (Model 4) or social capital (Model 5), the PRs of the no ME group were attenuated, but remained statistically significant. To check the effect of missing values in covariates and independent variables, we performed additional analyses that included missing data of each variable as a missing category. Although these re-analyses strengthened and partly altered the significant results, the main result that health behaviors had the greatest effect on impaired IA in the no ME group was unchanged (see Additional file 3).

**Table 3:** Prevalence ratios for impaired IA (IA score ≤ 3) associated with medical expenses

	Prevalence of impaired IA	Crude	Model 1^a^	Model 2^b^	Model 3^c^	Model 4^d^	Model 5^e^
		(*n* = 12,458)	(*n* = 12,305)	(*n* = 11,679)	(*n* = 11,498)	(*n* = 11,228)	(*n* = 11,318)
		PR (95% CI)	PR (95% CI)	PR (95% CI)	PR (95% CI)	PR (95% CI)	PR (95% CI)
Medical expenses in the past year							
Low	25.5%	1.00	1.00	1.00	1.00	1.00	1.00
Medium	25.1%	0.98 (0.91–1.06)	0.98 (0.90–1.05)	0.93 (0.86–1.00)	0.94 (0.87–1.01)	0.94 (0.87–1.02)	0.93 (0.86–1.01)
High	32.1%	1.26 (1.17–1.35)	1.16 (1.08–1.25)	0.97 (0.90–1.05)	0.99 (0.91–1.07)	0.96 (0.89–1.04)	0.98 (0.90–1.06)
None	32.5%	1.27 (1.16–1.40)	1.23 (1.11–1.35)	1.19 (1.08–1.31)	1.08 (0.98–1.20)	1.12 (1.01–1.24)	1.14 (1.03–1.26)
Health behaviors							
Health checks: no (ref: yes)					1.07 (1.003–1.14)		
Smoking: current (ref: never/ex-smoker)					1.28 (1.20–1.38)		
Frequency of exercise (h/w): < 1 (ref: ≥ 1)					1.46 (1.38–1.56)		
Dietary variety: low (ref: high)					1.27 (1.19–1.35)		
Oral health							
Use of extra cleaning devices: no (ref: yes)						1.16 (1.09–1.24)	
Bedtime brushing: not daily (ref: daily)						1.17 (1.10–1.26)	
Difficulty with chewing hard foods: yes (ref: no)						1.10 (1.02–1.17)	
Use of dentures: yes (ref: no)						1.02 (0.96–1.09)	
Social capital							
Social participation: no (ref: yes)							1.44 (1.35–1.53)
Social support: no (ref: yes)							1.19 (1.10–1.29)
Social networks: no (ref: yes)							1.28 (1.18–1.38)

PRs and 95% CIs were calculated using Poisson regression analyses

*CI* confidence interval, *IA* intellectual activity, *ref.* reference, *PR* prevalence ratio

^a^Model 1 is adjusted for age, gender, health insurance, accessibility to public facilities in their residential area, family size, and body mass index

^b^Model 2 is adjusted for the covariates in Model 1 plus physical and mental functioning (self-rated health, health-related QOL (PCS and MCS), sleep disturbance, depression, and cognitive functioning)

^c^Model 3 is adjusted for the variables in Model 2 plus health behaviors (health checks, smoking, frequency of exercise, and dietary variety)

^d^Model 4 is adjusted for the variables in Model 2 plus oral health (use of extra cleaning devices, frequency of bedtime brushing, difficulty with chewing hard foods, and use of dentures)

^e^Model 5 is adjusted for the variables in Model 2 plus social capital (social participation, social support, and social network)

## Discussion

Our study was the first to examine the association between a variety of factors and ME and reveal characteristics of the no ME group among community-dwelling elderly Japanese. The findings that community-dwelling elderly with no ME were less willing to cooperate with the survey, rarely had physical checkups, generally smoked and drank more often, did less exercise, had lower dietary variety, and practiced poor dental health behaviors suggested that they are less interested in their health and have unhealthier lifestyle behaviors, which could include not visiting doctors. Prior studies demonstrated that ME was significantly increased among obese people [15, 16] and reduced among people who spent a longer time walking [17]. Similar findings were confirmed in our study population, but lifestyle habits, including oral hygiene, were worst in the no ME group, with the exception of walking. A Germany study of adults aged 35 years or older [42] reported that higher health check attendance was associated with non-smoking, brisker physical activity, higher fruit and vegetable intake, and higher use of ambulatory care; not receiving health checks was thought to be a reflection of an unhealthy lifestyle choice, which supports our findings. Therefore, we propose that community-dwelling elderly with no ME require more intervention regarding their lifestyle habits.

We observed that the no ME group had the same degree of impaired higher-level functional capacity as the high ME group. An analysis of factors responsible for impaired IA suggested that impaired capacity in the high ME group could be explained by poor physical and mental functioning. The no ME group was high in physical and mental functioning but low in health-promoting behavior, and unhealthy lifestyle was identified as a possible determinant of impaired IA in this group. Many epidemiological studies have indicated that physical and mental functioning are strong contributors to higher-level functional capacity of the elderly [11–13, 38, 43, 44]. In particular, low IA is strongly associated with sensory impairment [38, 43] and a low grade of cognitive functioning [44].

Our findings that the no ME group had good physical and mental conditions but poor IA and social role are inconsistent with previous studies. One possible explanation for low IA in the no ME group is that in the TMIG-IC, the IA subscale includes reading and interest in health-related information [35]. Participants who were included in the no ME group and did not have healthy daily habits were considered to have lower IA because they were not interested in obtaining information about health and had fewer opportunities to read newspapers, books, and magazines. Regarding the mechanism underlying the association between health behaviors and IA, IA is a high-level life function represented by leisure activities, creativity, and searching [4, 35], and evaluation items of IA are classified into cognitively stimulating activities [39, 44]. Bad daily habits like smoking, having poor nutritional condition from consuming a smaller variety of foods, and missing regular exercise are connected to decreased oxygen and nutrition in the brain, which may cause the deterioration in their cognitive activities [24, 45]. This hypothesis is supported by prior prospective studies that reported an association of higher dietary variety [6, 22] and higher animal protein [7] with a lower risk of IA decline in older community-dwelling adults. However, this hypothesis is inconsistent with another prior study [5] that reported a significant association of current smoking and low frequency of exercise with the risk of low social role, but not with a decline in IA.

Our results showed that self-rated health became worse as ME became higher, which agrees with the findings from a previous study that regular visits to medical doctors were a prime determinant of self-rated health for Japanese community-dwelling elderly [46]. Because the no ME group had good health conditions and did not perceive themselves as being unhealthy, they had no reason to see medical doctors. However, the no ME group also had unhealthy lifestyle habits; even if they were not conscious of their poor health, there was a strong likelihood that they could contract lifestyle-related diseases but leave them untreated. It is therefore highly probable that community-dwelling elderly with no ME will have substantially greater needs for future hospitalization and continuous health care. Additionally, deterioration in IA can increase the risk of cognitive decline [44] and incident disability in BADL [11], which makes long-term care insurance necessary. A further prospective study is expected to confirm whether community-dwelling elderly with no ME have a high risk of augmenting near-future social security costs.

In our study, response rate showed a linear increase with age (see Fig. 1). Although prior studies failed to collect data on non-respondents [10, 47, 48], they suggested that younger people tended to respond to the questionnaire better, which is inconsistent with our results. A possible explanation for a lower response rate in younger age is that they are more involved in paid work than older people. Because working persons did not have enough free time, they may have been less likely to participate in our study than individuals without a job. Although we have no data on current working status, we think there is a high probability that job status may affect the response rate. Future studies are needed to verify whether the association between younger age and a lower response rate is observed in other age groups as well.

This study had some limitations. First, because our study was cross-sectional, we cannot confirm causal relationships. To overcome this limitation, a further prospective study should be performed. Second, nearly half of the community-dwelling elderly with no ME did not participate in this survey. This is consistent with a prior study reporting that people who had no healthcare utilization had lower levels of involvement in health surveys than people who had made at least one visit to the doctor [49]. This may have resulted in selection bias and requires special attention in the interpretation of the results. It is well-known that people who cooperate with health surveys are more interested in health and have better health behaviors and conditions compared to non-cooperators [50]. Therefore, lifestyle habits and higher-level functional capacity among the no ME group may have been underestimated. Third, our study’s sample was drawn from community-dwelling elderly aged 65–70 years. People of this generation were considered eligible because they could answer many questions more easily than people aged 70 years or older, and because there is a concern that they will have a major influence on social security costs in the near future. However, our findings on community-dwelling elderly above the age of 70 or elderly living in institutions should be interpreted with caution. Finally, we could not assess socioeconomic factors such as income and education, which have been shown to have effects on health care utilization [51], health behaviors [52], and IA [45]. Because the Japanese government offers health protection to economically poor persons through a welfare system, income may have an insignificant effect on physician visits, but education has the potential to be an important determinant of consultative behavior. Prior studies have demonstrated that lower educational attainment is associated with lower level of IA [38, 45]. Because our findings have a potential for confounding by educational background, future studies should consider education when evaluating the association between ME and IA among community-dwelling elderly.

## Conclusions

This cross-sectional study found that community-dwelling elderly with no ME had a low response rate, unhealthy lifestyle habits, and a low level of social capital. Higher-level functional capacity was diminished among both the high ME group and the no ME group. Our findings suggest that community-dwelling elderly who do not receive medical treatment were indifferent to health surveys and health-promoting behaviors, and undesirable health behaviors were a possible determinant of their impaired IA. However, further longitudinal studies are needed to explore the underlying causal relationships between ME, health behaviors, and IA among the community-dwelling elderly.

## Additional files

Additional file 1: Table S1. Characteristics of the study population responding to the questionnaire (*n* = 12,747). (PDF 102 kb)

Additional file 2: Figure S1. Percentage of participants with impaired capacity in each subscale of higher-level functional capacity according to medical expenses. (PDF 50 kb)

Additional file 3: Table S2. Prevalence ratios for impaired IA (IA score ≤ 3): Additional analyses with added missing data of each variable (*n* = 12,458). (PDF 72 kb)

## Abbreviations

BADL: Basic activities of daily living
BMI: Body mass index
CI: Confidence interval
IA: Intellectual activity
IADL: Instrumental activities of daily living
IPAQ-E: International Physical Activity Questionnaire Environment Module
MCS: Mental Health Component Score
ME: Medical expenses
PCS: Physical Health Component Score
PR: Prevalence ratio
QOL: Quality of life
SF-8: 8-item Short-Form Health Survey
TMIG-IC: Tokyo Metropolitan Institute of Gerontology Index of Competence
