# Supplementary material for: Cross-sectional association between medical expenses and intellectual activity in community-dwelling older adults
Source: Environ Health Prev Med. 2017 Aug 25;22:65. doi: 10.1186/s12199-017-0672-1 (PMC5664812; doi:10.1186/s12199-017-0672-1)
Supplement: Supplementary file 1 — Characteristics of the study population responding to the questionnaire (n = 12,747). (PDF 102 kb) [file 12199_2017_672_MOESM1_ESM.pdf]

Additional file 1: Table S1. Characteristics of the study population responding to the questionnaire (n = 12,747)

	Males (n = 5,413)		Females (n = 7,334)		P^{a}
	n	(%)	n	(%)	
**Demographics**					
Age at survey (years)					
65-67	2,439	(45.1%)	3,611	(49.2%)	<0.001
68-70	2,974	(54.9%)	3,723	(50.8%)	
Health insurance					
National Insurance	5,190	(95.9%)	7,131	(97.2%)	<0.001
Medical insurance for later-stage elderly	81	(1.5%)	28	(0.4%)	
Livelihood subsidies	142	(2.6%)	175	(2.4%)	
Family size					
1	599	(11.1%)	1,271	(17.3%)	<0.001
2	2,726	(50.4%)	3,837	(52.3%)	
3	1,214	(22.4%)	1,392	(19.0%)	
≥4	790	(14.6%)	771	(10.5%)	
Missing	84	(1.6%)	63	(0.9%)	
Body mass index					
Normal	3,920	(72.4%)	5,255	(71.7%)	<0.001
Underweight	243	(4.5%)	837	(11.4%)	
Obese	1,176	(21.7%)	1,167	(15.9%)	
Missing	74	(1.4%)	75	(1.0%)	

Additional file 1: Table S1. Continued

	Males (n = 5,413)		Females (n = 7,334)		P^{a}
	n	(%)	n	(%)	
**Health behaviors**					
Health checkups					
Present	3,636	(67.2%)	4,395	(59.9%)	<0.001
Absent	1,777	(32.8%)	2,939	(40.1%)	
Smoking habit					
Never-smoker	972	(18.0%)	6,121	(83.5%)	<0.001
Ex-smoker	3,082	(56.9%)	647	(8.8%)	
Current smoker	1,253	(23.1%)	387	(5.3%)	
Missing	106	(2.0%)	179	(2.4%)	
Number of years smoking					
0	980	(18.1%)	6,122	(83.5%)	<0.001
1-14	589	(10.9%)	292	(4.0%)	
15-29	865	(16.0%)	229	(3.1%)	
≥30	2,898	(53.5%)	536	(7.3%)	
Missing	81	(1.5%)	155	(2.1%)	
Frequency of alcohol consumption					
None	1,232	(22.8%)	3,802	(51.8%)	<0.001
Social	929	(17.2%)	1,857	(25.3%)	
Occasional	972	(18.0%)	981	(13.4%)	
Daily	2,227	(41.1%)	639	(8.7%)	
Missing	53	(1.0%)	55	(0.7%)	
Frequency of exercise (hours per week)					
≥5	1,275	(23.6%)	1,201	(16.4%)	<0.001
3-4	793	(14.6%)	1,212	(16.5%)	
1-2	1,185	(21.9%)	1,895	(25.8%)	
<1	2,065	(38.1%)	2,901	(39.6%)	
Missing	95	(1.8%)	125	(1.7%)	
Walking time (minutes per day)					
≥60	2,387	(44.1%)	3,269	(44.6%)	0.012
30-59	1,657	(30.6%)	2,384	(32.5%)	
<30	1,282	(23.7%)	1,578	(21.5%)	
Missing	87	(1.6%)	103	(1.4%)	
Dietary habits (dietary variety score)					
Low	2,624	(48.5%)	2,062	(28.1%)	<0.001
Medium	1,501	(27.7%)	2,317	(31.6%)	
High	1,208	(22.3%)	2,873	(39.2%)	
Missing	80	(1.5%)	82	(1.1%)	

Additional file 1: Table S1. Continued

	Males (n = 5,413)		Females (n = 7,334)		P^a
	n	(%)	n	(%)	
**Oral Health**					
Dental health behaviors					
Use of extra cleaning devices					
Yes	2,384	(44.0%)	4,515	(61.6%)	<0.001
No	2,819	(52.1%)	2,565	(35.0%)	
Missing	210	(3.9%)	254	(3.5%)	
Frequency of daily tooth brushing					
Three or more times	861	(15.9%)	1,998	(27.2%)	<0.001
Twice	2,396	(44.3%)	4,274	(58.3%)	
Less than twice	1,943	(35.9%)	808	(11.0%)	
Missing	213	(3.9%)	254	(3.5%)	
Frequency of bedtime tooth brushing					
Daily	3,471	(64.1%)	6,118	(83.4%)	<0.001
Sometimes	599	(11.1%)	581	(7.9%)	
Almost never	1,130	(20.9%)	380	(5.2%)	
Missing	213	(3.9%)	255	(3.5%)	
Oral function					
Having difficulty in chewing hard foods					
Absent	3,923	(72.5%)	5,729	(78.1%)	<0.001
Present	1,280	(23.6%)	1,347	(18.4%)	
Missing	210	(3.9%)	258	(3.5%)	
Choking on your tea and soup					
Absent	4,197	(77.5%)	5,704	(77.8%)	0.541
Present	1,000	(18.5%)	1,365	(18.6%)	
Missing	216	(4.0%)	265	(3.6%)	
Having dry mouth					
Absent	3,988	(73.7%)	5,409	(73.8%)	0.537
Present	1,204	(22.2%)	1,653	(22.5%)	
Missing	221	(4.1%)	272	(3.7%)	
Use of dentures					
Yes	2,184	(40.3%)	2,496	(34.0%)	<0.001
No	3,003	(55.5%)	4,547	(62.0%)	
Missing	226	(4.2%)	291	(4.0%)	

Additional file 1: Table S1. Continued

	Males (n = 5,413)		Females (n = 7,334)		P^{a}
	n	(%)	n	(%)	
**Social capital**					
Social participation (number of social groups)					
0	1,827	(33.8%)	1,969	(26.8%)	<0.001
1	1,524	(28.2%)	1,977	(27.0%)	
2	952	(17.6%)	1,495	(20.4%)	
≥3	1,019	(18.8%)	1,771	(24.1%)	
Missing	91	(1.7%)	122	(1.7%)	
Social support (number of people able to give emotional support)					
Four or more persons	703	(13.0%)	2,040	(27.8%)	<0.001
Two to three persons	2,280	(42.1%)	4,008	(54.6%)	
One person	1,603	(29.6%)	871	(11.9%)	
Nobody	667	(12.3%)	258	(3.5%)	
Missing	160	(3.0%)	157	(2.1%)	
Social networks (frequency of meeting friends)					
Nearly every day	663	(12.2%)	1,062	(14.5%)	<0.001
Several times per week	1,220	(22.5%)	2,389	(32.6%)	
Several times per month	1,495	(27.6%)	2,202	(30.0%)	
Several times per year	1,227	(22.7%)	1,039	(14.2%)	
Almost never	657	(12.1%)	485	(6.6%)	
Missing	151	(2.8%)	157	(2.1%)	
**Neighborhood environment**					
Access to public facilities					
Good	3,312	(61.2%)	4,724	(64.4%)	0.001
Poor	1,977	(36.5%)	2,471	(33.7%)	
Missing	124	(2.3%)	139	(1.9%)	
Access to public transport					
Good	4,819	(89.0%)	6,588	(89.8%)	0.245
Poor	462	(8.5%)	594	(8.1%)	
Missing	132	(2.4%)	152	(2.1%)	

Additional file 1: Table S1. Continued

	Males (n = 5,413)		Females (n = 7,334)		P^a
	n	(%)	n	(%)	
**Physical and mental functioning**					
Basic activities of daily living					
Good	5,224	(96.5%)	7,148	(97.5%)	0.006
Impaired	110	(2.0%)	104	(1.4%)	
Missing	79	(1.5%)	82	(1.1%)	
Self-rated health					
Very good	684	(12.6%)	1,001	(13.6%)	<0.001
Rather good	3,643	(67.3%)	5,143	(70.1%)	
Rather poor	658	(12.2%)	781	(10.6%)	
Very poor	292	(5.4%)	282	(3.8%)	
Missing	136	(2.5%)	127	(1.7%)	
Health-related quality of life (Physical Component Summary)					
High	1,860	(34.4%)	2,458	(33.5%)	0.602
Medium	1,686	(31.1%)	2,333	(31.8%)	
Low	1,758	(32.5%)	2,410	(32.9%)	
Missing	109	(2.0%)	133	(1.8%)	
Health-related quality of life (Mental Component Summary)					
High	1,848	(34.1%)	2,084	(28.4%)	<0.001
Medium	1,805	(33.3%)	2,600	(35.5%)	
Low	1,651	(30.5%)	2,517	(34.3%)	
Missing	109	(2.0%)	133	(1.8%)	
Sleep disturbance					
Absent	3,829	(70.7%)	4,814	(65.6%)	<0.001
Present	1,389	(25.7%)	2,251	(30.7%)	
Missing	195	(3.6%)	269	(3.7%)	
Depression					
Absent	3,987	(73.7%)	5,617	(76.6%)	<0.001
Present	1,266	(23.4%)	1,567	(21.4%)	
Missing	160	(3.0%)	150	(2.0%)	
Cognitive functioning					
Intact	4,396	(81.2%)	6,465	(88.2%)	<0.001
Poor	936	(17.3%)	783	(10.7%)	
Missing	81	(1.5%)	86	(1.2%)	

Additional file 1: Table S1. Continued

	Males (n = 5,413) n	(%)	Females (n = 7,334) n	(%)	P^{a}
**Higher-level functional capacity**					
Total score					
Good (score ≥12)	2,794	(51.6%)	5,383	(73.4%)	<0.001
Impaired (score ≤11)	2,449	(45.2%)	1,799	(24.5%)	
Missing	170	(3.1%)	152	(2.1%)	
Instrumental activities of daily living					
Good (score = 5)	4403	(81.3%)	6942	(94.7%)	<0.001
Impaired (score ≤4)	865	(16.0%)	267	(3.6%)	
Missing	145	(2.7%)	125	(1.7%)	
Intellectual activity					
Good (score = 4)	3510	(64.8%)	5454	(74.4%)	<0.001
Impaired (score ≤3)	1748	(32.3%)	1746	(23.8%)	
Missing	155	(2.9%)	134	(1.8%)	
Social role					
Good (score = 4)	1913	(35.3%)	4316	(58.8%)	<0.001
Impaired (score ≤3)	3338	(61.7%)	2874	(39.2%)	
Missing	162	(3.0%)	144	(2.0%)	

[a]Differences between males and females were analyzed using Fisher's exact test.
