# Supplementary material for: Cross-sectional association between medical expenses and intellectual activity in community-dwelling older adults
Source: Environ Health Prev Med. 2017 Aug 25;22:65. doi: 10.1186/s12199-017-0672-1 (PMC5664812; doi:10.1186/s12199-017-0672-1)
Supplement: Supplementary file 2 — Percentage of participants with impaired capacity in each subscale of higher-level functional capacity according to medical expenses. (PDF 50 kb) [file 12199_2017_672_MOESM2_ESM.pdf]

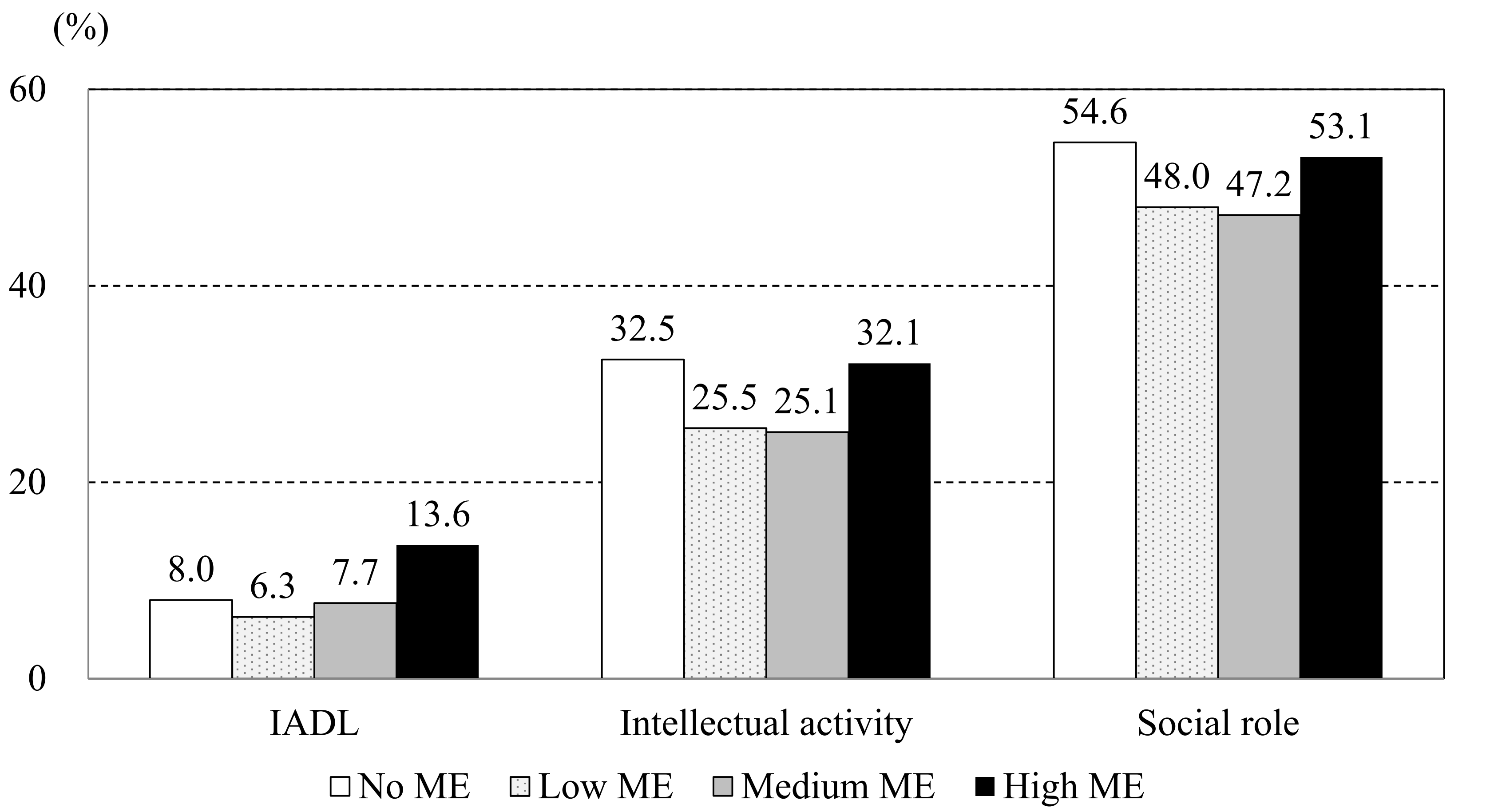

Additional file 2: Figure S1. Percentage of participants with impaired capacity in each subscale of higher-level functional capacity according to medical expenses. *IADL* instrumental activities of daily living, *ME* medical expenses
