# Supplementary material for: Cross-sectional association between medical expenses and intellectual activity in community-dwelling older adults
Source: Environ Health Prev Med. 2017 Aug 25;22:65. doi: 10.1186/s12199-017-0672-1 (PMC5664812; doi:10.1186/s12199-017-0672-1)
Supplement: Supplementary file 3 — Prevalence ratios for impaired IA (IA score ≤ 3): Additional analyses with added missing data of each variable (n = 12,458). (PDF 72 kb) [file 12199_2017_672_MOESM3_ESM.pdf]

Additional file 3: Table S2. Prevalence ratios for impaired IA (IA score ≤3): Additional analyses with added missing data of each variable (n = 12,458)

	Model 1[a] PR (95% CI)	Model 2[b] PR (95% CI)	Model 3[c] PR (95% CI)	Model 4[d] PR (95% CI)	Model 5[e] PR (95% CI)
Medical expenses in the past year					
Low	1.00	1.00	1.00	1.00	1.00
Medium	0.98 (0.90-1.05)	0.92 (0.86-0.99)	0.94 (0.87-1.02)	0.93 (0.86-1.001)	0.93 (0.86-0.999)
High	1.16 (1.08-1.25)	0.97 (0.90-1.05)	1.00 (0.93-1.07)	0.98 (0.91-1.05)	0.98 (0.91-1.05)
None	1.24 (1.12-1.36)	1.21 (1.10-1.33)	1.11 (1.01-1.22)	1.16 (1.06-1.28)	1.15 (1.05-1.26)
Health behaviors					
Health checks (ref: participation)					
Non-participation			1.07 (1.004-1.14)		
Smoking (ref: never or ex-smoker)					
Current			1.27 (1.18-1.35)		
Missing data			1.04 (0.85-1.27)		
Frequency of exercise (ref: ≥1 hour a week)					
<1 hour a week			1.46 (1.37-1.54)		
Missing data			1.28 (0.92-1.77)		
Dietary variety (ref: high)					
Low			1.27 (1.20-1.35)		
Missing data			1.32 (0.93-1.87)		
Oral health					
Use of extra cleaning devices (ref: yes)					
No				1.16 (1.10-1.23)	
Missing data				1.51 (0.98-2.33)	
Bedtime brushing frequency (ref: daily)					
Not daily				1.19 (1.12-1.27)	
Missing data				0.86 (0.57-1.29)	
Having difficulty with chewing hard foods (ref: no)					
Yes				1.09 (1.02-1.16)	
Missing data				0.93 (0.62-1.41)	
Use of dentures (ref: no)					
Yes				1.03 (0.97-1.09)	
Missing data				1.09 (0.83-1.44)	
Social capital					
Social participation (ref: yes)					
No					1.43 (1.35-1.52)
Missing data					1.16 (0.93-1.45)
Social support (ref: yes)					
No					1.19 (1.10-1.28)
Missing data					1.13 (0.88-1.45)
Social networks (ref: yes)					
No					1.28 (1.19-1.37)
Missing data					1.07 (0.84-1.37)

CI confidence interval, *IA* intellectual activity, *PR* prevalence ratio

[a]Model 1 is adjusted for age, gender, health insurance, accessibility to public facilities in their residential area, family size, and BMI.

[b]Model 2 is adjusted for the covariates in Model 1 plus physical and mental functioning (self-rated health, health-related QOL (PCS and MCS), sleep disturbance, depression, and cognitive functioning).

[c]Model 3 is adjusted for the variables in Model 2 plus health behaviors (health checks, smoking, frequency of exercise, and dietary variety).

[d]Model 4 is adjusted for the variables in Model 2 plus oral health (use of extra cleaning devices, frequency of bedtime brushing, difficulty with chewing hard foods, and use of dentures).

[e]Model 5 is adjusted for the variables in Model 2 plus social capital (social participation, social support, and social network).
